# The role of cilia and ciliary motility in otolith formation in the zebrafish embryo

**DOI:** 10.1186/2046-2530-1-S1-P78

**Published:** 2012-11-16

**Authors:** G Stooke-Vaughan, P Huang, KL Hammond, AF Schier, TT Whitfield

**Affiliations:** 1 University of Sheffield,UK; 2 Harvard University, USA

## 

Zebrafish otic vesicle (OV) luminal cells are monociliated from cavitation at 18.5 hours post fertilisation. Over the next 6 hours, otolith precursor particles are secreted into the OV and tether to the tips of the first forming hair cell kinocilia at the anterior and posterior poles of the OV, forming two otoliths (structures required for hearing and balance). Previous models of the mechanism of otolith tethering have recognised the importance of cilia for this process but disagreed on the number and distribution of motile and immotile cilia in the OV. Using high-speed video microscopy we have shown that the majority (92-98%) of OV cilia, including the kinocilia, are immotile. Motile cilia are concentrated at the poles of the OV near the forming otoliths, and a few motile cilia are present on the medial wall of the OV. Mutants with a reduced number of cilia (*iguana*) or ciliary motility defects (*lrrc50*) often have abnormal otoliths. We have also shown that hair cells are required for otolith nucleation: if ectopic hair cells are formed (*mindbomb* mutant or *Su*(*H*) morphant), otolith precursor particles bind to all the kinocilia. If hair cells are absent (*atoh1b* morphant), otolith formation is delayed, and a single untethered otolith forms. Surprisingly, in the absence of cilia (*MZovl* mutant) otolith precursors can tether on the apical surface of hair cells, including ectopic hair cells formed in an *MZovl* mutant injected with *Su*(*H*) morpholino. Embryonic movement also plays a minor role in normal otolith formation.

http://cdbg.shef.ac.uk/research/whitfield/

